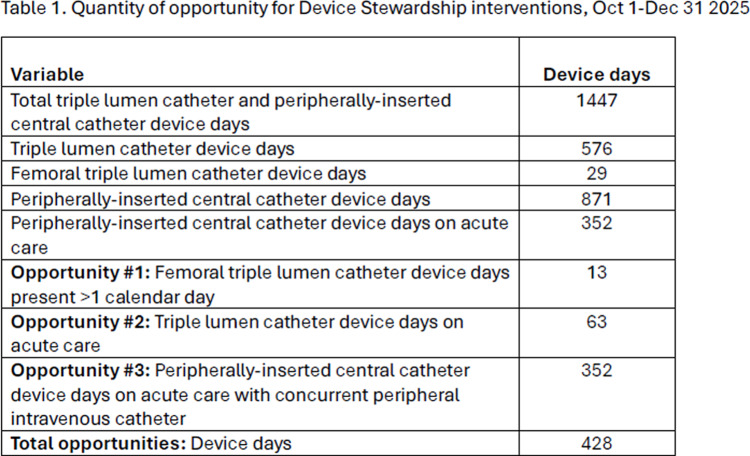# 163 The Utility of Follow-Up Blood Cultures in Gram-Negative Bacteremia

**DOI:** 10.1017/ash.2026.10564

**Published:** 2026-06-23

**Authors:** Rohana Bruker, Marlee Barton, Jacob McAlinn, Angela Montgomery, Guillermo Rodriguez Nava, Heather Young

**Affiliations:** 1 Denver Health Hospital Authority; 2 Denver Health and Hospital Authority; 3 Denver Health; 4 Denver Health Medical Center

## Abstract

**Background:** Vascular access devices (VAD), including central venous catheters (CVC) and peripheral intravenous catheters (PIVC), are among the most common medical intervention for hospitalized patients, yet they are also a major source of infectious and noninfectious complications. The Centers for Disease Control and Prevention (CDC) recommends prompt removal of CVC that are no longer indicated as a cornerstone of CLABSI prevention, although cessation of CVC indication is ill-defined. Our hospital implemented a Device Stewardship program to identify VAD that were likely inappropriate and to nudge providers to remove them promptly. The objective of this study is to quantify the opportunities to decrease VAD utilization and to describe early Device Stewardship efforts. Methods Study design and population. This is a retrospective cohort study at a 500-bed academic safety net hospital in Denver, CO. All inpatients hospitalized between 10/1/2025 and 12/31/2025, were eligible for inclusion. CVC placed for ECMO or rapid rewarming were excluded. Definitions. The following VAD were generally considered unnecessary: Femoral triple lumen catheters (TLC) in place for <1 day TLC present in a patient on an acute care unit Duplicate VAD (peripherally-inserted central catheter [PICC] and PIVC) in a patient on an acute care unit Intervention. Infection preventionists (IPs) identified eligible patients through an Epic worklist. Standardized text messages were used to suggest de-escalation of VAD to nursing and provider teams on weekdays (Figure 1). Results There were 576 TLC device days and 871 PICC device days in hospitalized inpatients during the study period. VAD selection could be improved in 29.6% of these patients (Table 1). Duplicate VAD was identified as the most frequent opportunity for improvement whereas femoral TLC present for <1 day was the least frequent opportunity. IPs contacted the clinical teams of 64 unique patients with duplicate VAD to suggest removal of ≥1 PIVC; 36 PIVC in 25 patients (39%) were removed as a result of intervention. Additionally, 5 femoral TLC present for <1 day and 15 TLC on acute care units were intervened upon with 1 femoral line and 3 TLC (20% each) removed during the study period. Conclusion Despite national guidelines to remove unnecessary VAD, there is ample opportunity to deescalate VAD in clinical practice. Simple criteria can be developed to standardize the selection and to prompt earlier removal of VAD, particularly duplicate access. Work can be done to improve the acceptance rate of suggestions.

**Figure:**
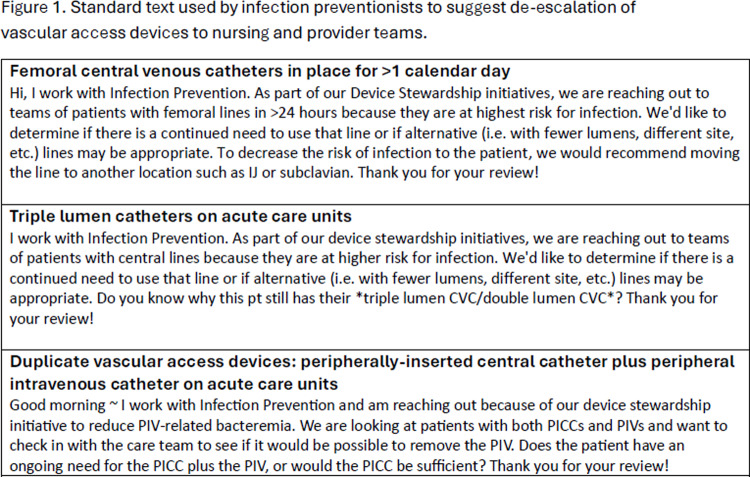

**Figure:**